# The Hospital Nursing Supplement

**Published:** 1893-08-26

**Authors:** 


" The Hospital" Nursing Mirror.

Being the Extra Nursing Supplement of "The Hospital" Newspaper.

[Contributions for this Supplement should be addressed to the Editor, The Hospital, 428, Strand, London, W.C., and should have the word "Nursing" plainly written in left-hand top corner of the envelope.]

### News from the Nursing World.

### NURSING SISTERS.

The lady members of the St. John Ambulance Brigade do a good and useful work. They made themselves specially of service on the Royal wedding day, when between one and two thousand cases of injuries and fainting were attended to by the corps which they assisted. These Nursing Sisters give their services freely for the benefit of the sick and suffering of the metropolis, and on all occasions of public gatherings are to the fore, with all the ambulance paraphernalia of tents and waggons. The qualifications for a Nursing Sister of this Brigade is that she must hold a "First Aid" certificate, and she is then entitled to attend the teaching on more advanced nursing which is given at the headquarters of the Brigade every fortnight. All particulars may be had from the Hon. Secretary, Mrs. J. Calvin Lines, 45, Wharton Road, W.

### CLOTHING FOR INFECTIOUS HOSPITALS.

It not infrequently occurs that an experienced nurse, or even matron, placed in charge of a new isolation hospital, or department of a hospital, is met with the difficulty of providing accessories totally different to those in use in general nursing. Questions which are sent to us on the subject prove that it is so. Quite recently a correspondent wrote to us inquiring as to the material used for suits for male patients in the Asylums Board hospitals. It is certainly wise to use, what has been tested as suitable and durable rather than to experiment in such a direction. For the use, therefore, of others, we mention here that the material used is a light blue washing serge, and this is used not only by the Asylums Board authorities, but in the army and naval institutions also.

### WORKHOUSE INFIRMARY NURSING.

That reforms in workhouse infirmary nursing are sorely needed, is abundantly proved. Further evidence came out recently at an inquiry held upon the death of an aged pauper in the Fulham Infirmary. It is stated that this poor old man, who had fallen out of bed, was seven hours without medical help. On enquiry it appeared that the nurse in charge had two wards, some 57 sick people in all, under her care, with the natural consequence that it was impossible for proper attention to be given to any of them. We hear a great deal as to the coming necessity of rate-supported ho spitals and charities, but the voluntary system does not give ground for such scandals as these, and if rate-supported institutions are to be managed on the lines of the Fulham Infirmary, it will be indeed a bad day for this country when the voluntary system has become a thing of the past.

### VOLUNTARY AND INVOLUNTARY ALMS.

Under the guise of "charity" many meannesses are perpetrated, almost tempting us to believe that the original meaning of the beautiful word has been forgotten. People part with various old articles under the misapprehension that what is worthless to themselves increases mysteriously in value during its transit to our poorer brethren. These are the gifts which cost us nothing, but it needs kindly sympathy and intimate acquaintance with recipients to make offerings of benefit alike to givers and receivers. Hospital patients are as keen to detect and appreciate the difference as the shrewdest of lawyers. Children's spontaneous gifts are often precious from the lavish and ungrudging method of bestowal. Little folks in luxurious homes have learnt one of the best lessons in life when they begin to share their blessings with the waifs and strays of humanity. Many a healthy party of youngsters nowadays has its own particular little invalid located, by means of their private contributions, in some seaside home. Their own holidays lose none of their zest because of this act of loving kindness. But children's acts of generosity must be done willingly, for unless they take joy in the doing there is no grace in the deed. The following passage appeared in a report of a foreign mission, and most readers must doubt there being anything voluntary about the self-denial of these "mitherless bairns." But children's charity should be voluntary, otherwise it is of little worth, and gifts obtained from an orphanage—in the way recently stated in the missionary magazine—will cause mothers at any rate to sympathise pitifully with the small donors; the wording is, "From seven little orphans, by denying themselves butter and jam for the week, 4s." Poor wee orphans!

### CLAPHAM MATERNITY HOSPITAL.

From the committee of the Clapham Maternity Hospital comes an urgent appeal for £25,000 for much needed additions to the present building. Until now the committee have rented 41, Jeffrey's Road, but the work has so greatly increased and developed that it is absolutely necessary to enlarge the hospital premises, and an opportunity now occurs for the purchase of the present house and the one adjoining—they are semi-detached—and turning them into one building. Furniture will also be needed for the additional house, to be provided from the £25,000 asked for. We hope the necessary funds may speedily be forthcoming.

### HINTS.

Few people give enough thought to their eyes—to the care of them, at any rate—and surely this neglect is almost as harmful as definite ill-usage, and examples of the latter also come every day within our own experience. In the railway carriage, if an unlucky person gets a morsel of grit in his eye, his sufferings from the intrusive atom are considerably augmented by the remedies with which all the other passengers assail him. Or a fly gains an entrance, and some well-meaning friend inflicts small tortures on the victim in endeavouring to dislodge the tiny creature. Could not some, at any rate, of these pains, and possible injury, to a most beautiful and valuable organ be avoided by a little common sense? If every district nurse and every trained woman lecturer took all available opportunities of pointing out danger, and inculcating safe and simple remedies, much needless discomfort would be avoided.

### WORKHOUSE NURSING AT LEEDS.

We are always glad to welcome any effort in the direction of the much-needed reforms now being carried on in so many parts of the country in the matter of workhouse infirmary nursing, but we are inclined to think there is a happy medium in the methods to be adopted by workhouse authorities for the furtherance of this object, and those members of the Leeds Board of Guardians who took exception to the large sum which was said to be needed for the erection of a nurses' home in connection with the union workhouse, have possibly some right and reason on their side. Eight thousand pounds is the sum which will be required for the Home, in which accommodation for forty nurses will be provided, if the differences which are at present disturbing the Guardians, on the question of cost, are happily settled; but the sum is undeniably a large one, and it might surely be possible for improvements of a less expensive nature to pave the way for further reforms when times are better and money more plentiful.

### LOW FEES AND INEFFICIENCY.

No sooner has the public fully acknowledged the superiority of the trained nurse of the present day, than they seem ready to lower the standard, and the cry is now for nurses with less training and lower fees. This is especially the case in respect to the parish nurse, who above all should, according to common-sense, possess experience and reliable training to meet the emergencies of her calling. In one district —Hailsham—the cry is for a Ladies' Nursing Asso. ciation to instruct the wives of the working classes in the art of nursing. Such an association would be doubtless very useful in giving practical teaching in simple nursing through the medium of a thoroughly trained teacher, but we cannot agree with the chairman of the Board of Guardians at a recent meeting at Hailsham, who said that he thought many women might be found in the neighbourhood who would be capable of discharging the duties of nurse for a smaller fee than a "professional nurse" required, provided they had the "necessary training." He referred to such training as could be supplied by the Ladies' Association, and not to the practical and thorough instruction so necessary, and only possible through hospital experience.

### NURSING AT LYNN.

Not very long ago an institute for private nurses was added to the West Norfolk and Lynn Hospital. We are glad to learn that the scheme has proved a success in every way, and has attracted much interest in the neighbourhood. A sale of work has just been held in aid of the institution, which was admirably supplied by the gentry and tradespeople of the district, the goods meeting with a ready sale. The proceedings were opened by Lady Ffolkes, and several speeches in warm approval of the scheme were made.

### THE FIRST CERTIFICATE.

A Correspondent sends us information to the effect that "Great rejoicings were held at St. Luke's Home, Vancouver, last month, when Nurse Creighton received the first certificate issued by the Home, from the hands of Bishop Sillitoe. This certificate was supplemented by a testimonial from the Bishop himself, testifying to the appreciation in which Miss Creighton's work was held at the Home, Convalescent Home, and amongst private patients. Altogether the occasion was a most gratifying one for St. Luke's, and Sister Francis celebrated the event further by a reception at the Home, which was gaily decorated."

### NURSES AS MISSIONARIES.

That is an excellent idea, which originated we believe in America, of sending out nurses as missionaries. Healing the sick formed part of the early Christian evangelizing, and doubtless those services of love were not the least powerful aids of the Apostles. The Zenana Mission promises to become an increasing agent for good, which is only possible through the agency of medicine. What a useful addition a missionary nurse would be on any mission station. This would afford another noble and useful opening for women, and should especially commend itself to some of those women who, whilst longing to enter the field of labour, feel scruples about filling remunerative posts, more needed by others.

### THE INDIAN NURSING SERVICE.

Sixty days' privilege leave of absence, from July 20th, is granted to Deputy Superintendent, Miss M. J. Hislop, Indian Nursing Service, attached to the Station Hospital, Allahabad. Under the provisions of the regulations relating to the Indian Nursing Service at present in force, the Principal Medical Officer, her Majesty's Forces, in each presidency is empowered to grant privilege leave of absence not exceeding sixty days in the year to a lady nurse, on the understanding that the State is put to no extra expense by its being granted. The Government of India has decided that the above ruling should be modified in order to conform to the practice observed in the case of applications for grant of privilege leave from other officers, and has directed that privilege leave to lady nurses may in future be granted by General Officers Commanding Divisions or Districts, but moderation is to be exercised in the grant of this indulgence, care being taken that the efficiency of the service may not be impaired by there being a paucity of ladies of the Indian Nursing Service.

### SHORT ITEMS.

The Westport Guardians, Ireland, seem to have got into a strange difficulty in an unaccountable manner. They advertised for a nurse, but pending the appointment the guardians have repented them, and have come to the conclusion they don't want a nurse after all, and that there are no fever cases for her to nurse.—The St. Lawrence Home for providing trained nurses for the sick poor has had its hands full during the past month, the four nurses on the staff having made 2,935 visits.—A collection in aid of the Village Nurse Fund was held last Sunday at St. John's Church, Brighton. It was no short-sighted policy of the Newtown Guardians to make a small grant towards the maintenance of a parish nurse, since attention such as a good nurse can give often saves many from burdening the rates.—The Nurses of the Manchester and Salford Sick Poor Association paid 28,963 visits during the last quarter.—The Nursing Institute at the Homœopathic Hospital certainly earns a very considerable income. In 1892—the influenza year—the earnings amounted to £2,507, whilst last year, though considerably less came in, the sum, £1,732, was by no means inconsiderable.—The demand for classes for instruction in nursing is increasing all over the country, and it would appear that it will soon become a definite item in the programme of technical education.

### Notes of a Lecture on Cholera.

*Delivered by Mrs. Scharlieb, M.D.*

By One of the Audience.

Up to the beginning of the present century cholera was only found in Asia (Asiatic cholera), especially India. The Valley of the Ganges, where the ground is mostly marshy, is probably the source of the universal dissemination of this disease, for when the Ganges floods, it scatters the germ over the adjacent low-lying lands. The first European epidemic began in 1826 in Lower Bengal; thence it travelled to Khiva in 1829. In the beginning of 1831 it reached St. Petersburg; at the end of that year it was found in the Medway, and in 1832 in New York. Later epidemics pass more rapidly from place to place, owing to increased travelling facilities as furnished by steamboats, the railway, &c., in contradistinction to the earlier method of travelling by caravan.

The comma bacillus is the true germ of cholera. The micro-organism is rod-shaped, and curved, its length is from 1½ to 2½ micro-millimetres, and the width is half a micro-millimetre; it multiplies by fission. This germ may be conveyed by water, milk, and all articles contaminated by cholera evacuations. When cholera excreta are left to dry exposed to the atmosphere, the germ is disseminated through the air. The bacillus is killed by boiling, but not by alcohol, as is often thought. As a matter of fact, alcoholic individuals who take alcohol are generally the most susceptible. The bacillus contained in the evacuations can be destroyed by mixing with an equal part of any of the following: (*a*) 5 per cent. solution of carbolic acid; (*b*) 2 per cent. solution of perchloride of mercury; (*c*) 4 per cent. solution of perchloride of lime; (*d*) 5 per cent. solution of sulphate of iron.

Steamers should be purified by steam passed through under pressure.

All suspected clothing and bedding should be destroyed if possible—if not, they should be steamed under pressure for one hour, or boiled at least twenty minutes, then immersed in perchloride of mercury 2 per cent. solution for twenty-four hours, and at the end of that time thoroughly aired.

The vomit and dejections should be mixed with strong solution of corrosive sublimate (2 per cent.) All stained articles should be plunged into one of the above-mentioned strong solutions for at least four hours. Water and milk must be boiled. The floors and walls of hospitals and houses should be washed with disinfectant—viz., 5 per cent. carbolic acid solution.

Latrines should be disinfected twice daily, using one of the above-mentioned strong solutions in quantity equal to the amount of dejecta contained.

All doctors and assistants nursing cholera patients should wear overalls, to be taken off on leaving the sick-room. Plates, dishes, jugs, cups, &c., are to be washed in disinfectant, and purified over a spirit flame. The evacuations should be removed as speedily as possible, as when fresh they do not possess the same virulence as when they are left for some time.

The dead should be wrapped in a sheet saturated with carbolic, and then placed in a coffin surrounded by chloride of lime. If possible, it is better that the body should be cremated at once.

The following influences predispose to cholera:—

1. Any cause tending to lower the vitality at the time, *e.g.*, fear, overwork, chills, diarrhœa, and indigestion, which also cause inflammations; and lesions, which form a suitable nidus for germs to implant themselves.

The greatest source of infection is water. This may be infected at its source, in its transit, through adulteration of milk by water, or through vegetables and fruits. A great deal of the vegetables and fruit of the London markets is imported from Marseilles, where cholera is practically always found. The gardeners of Marseilles as a rule use the evacuations mixed with water to fertilise the soil, so that this is a dangerous source of cholera. As a safeguard it is well to always wash lettuce, &c., with salt and water before using, but it will be wiser to take no green vegetables unless home grown.

The incubation period for cholera ranges from 48 to 60 hours. Cholera may occur in mild or abortive attacks or as a malignant disease. There is generally some premonitory diarrhœa.

During the stage of invasion, the stools, which may occur as frequently as 50 per diem, but often less, acquire a typical appearance, which has been compared to rice water. In this stage vomiting occurs, first of food, and later of rice water material. The patient is troubled with acute pain in the back, stomach, and abdomen, with cramps in the limbs.

In the stage of collapse the skin is inelastic and the surface of the body cold. The hands and feet are corrugated, the cheeks fall in, the eyes are sunken in the head, the breath is cold, the voice husky and whispering or even quite inaudible. The purging and vomiting are less frequent, but the patient gradually becomes worse, and supression of urine occurs. During the stage of collapse the patient may die, if not, the stage of reaction sets in. The countenance becomes more natural, the complexion loses its leaden hue, the colour returns to the evacuations, and restitution of the renal function occurs; this is the best sign of commencing convalescence.

The stage of collapse in some very rare cases may proceed to cholera typhoid, which is marked by high fever and a typhoid state.

Treatment.—1. Premonitory Diarrhœa. This is a painless diarrhœa. The patient should be at once placed in bed with hot bottles, a mustard plaster to the abdomen, kept in its place by a flannel belt.

The diet should consist of water or milk arrowroot, with about a dessert spoonful of brandy occasionally. Chicken or veal broth may be given. The patient should not be allowed to take beef-tea, because in this salines are present as extractives, and these would tend to increase the diarrhœa.

A good mixture is the following: ℞ Acid sulph. aromat., ♏x. to xv.; tr. opii, ♏x. to xv.; ac. carbolic, ♏i.; aqua cinnamoni, ad. ℥p.; one tablespoonful T.A.S.

During the stage of invasion give the same acid mixture or the cholera saline, *e.g.*, ℞ Sodii chloridi, grs. xv.; potassii chloridi, grs. x.; Sodii hypophosphitis, ℥p.; aqua anethi, ad ℥p.; one tablespoonful thrice daily.

Should the patient be restless, chloral, grs. x. may be added. In collapse nothing should be given by the mouth. The patient should be kept warm. The foot of the bed should be elevated.

A hypodermic injection of ether or ammonia or caffeine and salicylic acid may be given, and in severe cases transfusion of blood or of normal saline solution may be performed.

### A Useful Association.

The truth of the adage, "Still waters run deep," constantly asserts itself, and we are often surprised to hear of the work of societies whose name even is unfamiliar, because they work rather than advertise. Such an institution is the Women's University Settlement in Southwark for training ladies in philanthropic work, and the latest branch of which is the "Benson Home" for District Nurses. Many people think that district visiting requires no more previous knowledge than the afternoon call of society—that is to say, until they have tried it. The clergy, however, and those who have to direct affairs amongst the poor, know that there are few things more difficult than to find any number of competent workers. Miss Sewell, the Warden of the Settlement, fully recognises this, and there can be no doubt that she will confer a lasting benefit both on the workers and the poor themselves, by this excellent scheme.

### On "Giving In."

By a Nurse.

Some weeks ago an article appeared in The Hospital headed "Why Do Probationers Break Down?" Medical officers and superintendents of nursing called it "very good," but nurses and probationers said, "Yes, that's all very true, but we wish the writer had explained at what stage we ought to give in, that's our difficulty."

Yet that is the special point on which no rule can be laid down; it must rest with the weary worker, and she must earn to judge for herself. To give in for every trivial pain or weakness is not to be thought of by any earnest woman. Moreover, experience proves how easy it is to bear a slight trouble or discomfort when living amongst men, women, and children in hospital wards and seeing their patience, nay cheerfulness, under grave sufferings.

The weakly, poor-spirited woman who "gives in" constantly, and literally "enjoys" her ill-health, has no business amongst invalids, she has no right to pose as a worker amongst the sick poor; it is not fair to them or to herself or to her friends outside the hospital.

But conscientious persons, whose average health is good, ought never to take advantage of that fact to overstrain their powers of endurance. They possess a treasure which they ought to guard wisely, for sound health is beyond and above all other good things. It makes life worth living, and it makes work enjoyable. Good temper and even spirits generally accompany fine health, and certainly they ought always to do so.

Therefore let strong women husband their strength, and when they feel it is beginning to fail under the stress of ward work and anxiety let them own up boldly that this is the case. Perhaps twenty-four hours absence will put things right, if the time be wisely spent, in a different scene, in fresher air, and in congenial society.

It requires a moral effort to say one is tired, or seedy, or depressed, but each worthy worker who bravely takes this step, benefits not only herself, but her fellow nurses. She helps to create a desirable precedent, and this should reward her for the possible coldness with which her confession is received at headquarters.

The strictest of disciplinarians could not refuse to grant leave which was so reasonably requested, and many a matron would reward the petitioner with prompt and sympathetic acquiescence. Of course it is not always so; we are all acquainted with hard folks who condemn everybody's weakness save their own; and to such as these their probationers go trembling, and only at the moment when a simple need for "giving in" is threatening to become a serious case of breaking down.

If confessions of weariness or weakness were always received with the kindness which is their due, we should cease to hear, "Oh, I could not think of saying I feel out of sorts, it is no good to risk being snubbed. I'll wait a day or two, and if I am ill, then I know everybody will be patient and kind enough." There is one matron we know whose nurses say they never have a chance of getting ill, they are too well looked after; and another matron writes to her probationers when they are having their annual holiday and bids them let her know truthfully whether they are quite well and perfectly rested before she fixes the date of their return. We are inclined to think such heads of nursing schools are better served and infinitely more respected than their less merciful sisters.

To put it on the lowest grounds, it is economy in the long run to conserve the health of working bees; the drones usually take unnecessary care of themselves, but the useful people always need encouragement to indulge in a reasonable amount of "giving in."

### Nurses' Homes.

### II.—PRESENT.

The nursing ranks are now so frequently recruited from the educated classes, and there is such an increase in the rate of payments and in the facility for obtaining work, that a demand has been created for better accommodation for private nurses. The strain upon the mental and physical powers of those engaged in the care of the sick is far greater than it formerly was, and nurses return from cases with tired bodies and exhausted nerves.

Certainly we find in nursing institutions connected with hospitals, and also those kept by private individuals, that the demand for more comfort has been thoroughly responded to. The underground sitting-room, with its dingy furniture has almost disappeared, and light and comfortable apartments have been provided for the staff. Couches, books, plants, and usually a piano, combine to make these rooms pleasant retreats for tired bodies and brains. The food, though plain, is good and sufficient, and generally well cooked and served. To secure these comforts without any exertion on their own part, many nurses are willing to give up a large portion of their earnings.

But there remains the large and increasing number of those who wish to work for themselves and to find their own homes. The more independent and energetic prefer to take a small room, at a rent of £12 to £15 per annum, and to furnish it after their own tastes, the people of the house or a fellow-lodger agreeing to supply the attendance required and to answer the bell. Many of these little homes are exceedingly dainty and pleasant, and a nurse who prefers liberty and quiet is wise in living alone, though she must spend about one-fifth of her income in rent and extras. Sometimes a number of nurses combine to take a flat, and to pay a housekeeper or "mother," who looks after their home interests. These little communities consist of the most practical and progressive members of the profession, who are keenly alive to the advantages of co-operative housekeeping.

On the other hand, the nurse who prefers furnished lodgings apparently finds it difficult to secure the kind of home she requires. For social and sanitary reasons she must live in a good class of house, and her expenses are proportionately high. Either she must be willing to pay one-fourth of her income as rent for a room to be kept always ready for her, or she must take the chance of finding her usual quarters occupied and face the difficulty of hunting for others. Landladies also are sometimes the reverse of kindly. The good-tempered ones are apt to be slovenly about their houses and persons, and careless in answering the bell, while the smart, neat mistress of the house is often narrow, both in views and ways; she has an objection to women lodgers, and keeps a suspicious eye upon their movements.

It is quite true that the landlady has also her grievances. Nurses are not profitable inmates. There is nothing to be made out of the food supplied to them, for, unless they agree to board by the week, economy is always practised in this direction. The large boxes that accompany them from case to case injure the staircase in their frequent ascents and descents on the back of the hired man, who is usually some one not industrious or healthy enough to obtain regular employment. Again, the nurse is liable to be summoned in the night, and the whole house is sometimes aroused by an impatient attack on the knocker and bell.

To avoid these difficulties a large number of nurses prefer to live in hostels and homes established solely for their convenience. If the nurse is content to sleep in a cubicle at the ordinary charge of a shilling per night, this is decidedly the most economical way of living; if she prefers to rent a room the charge per week is much the same as outside. There are, however, great advantages to be gained, such as a general sitting-room, meals on the premises, inquiries answered, &c. The usual tariff is as follows: Breakfast, 6d. to 9d.; lunch or supper, 9d.; dinner, 1s.; tea, 4d. to 6d. A small extra charge is made for the storage of boxes, which, containing, as they often do, the accumulations of years, are most difficult to dispose of in the limited allotted space. Those who are practically acquainted with house-rents and taxation will see by the above quotations that, unless these homes are frequented by a large number of nurses it is almost impossible for them to pay their way. Some of the proprietors recognizing this fact eke out their incomes by letting rooms to paying patients, in fact, combine a medical home with the nurses' home, an arrangement obviously bad for the latter. Others restrict themselves to sending out young nurses to whom they pay a somewhat small salary, receiving their earnings in return. One or two have pluckily depended on the patronage of trained nurses alone, but their books are likely to show a decided balance on the wrong side.

Unpractical nurses are apt to complain of the charges made at these establishments and to point out that homes for working women can be managed at less expense. As a matter of fact the daily governess and the girls employed in shops are regular boarders from one month's end to the other, and can therefore be provided for at less cost than the nurse, who comes in for short periods at irregular intervals. They are also apt to forget that though the cost price of food is very little, service, coals, and wear and tear are costly items which double the expenditure, and that while a shilling may seem a fair rent for a cubicle, yet if the bed is only occupied one night there is very little profit, after the washing of sheets, pillow slip, and towel, is paid. Most perfect management is required to make both ends meet, and at the same time to satisfy the demands of the nurses. The list below will give an idea of the average house expenditure of these places; it is copied almost verbatim from the accounts of a well-known home: Rent, £130; taxes and rates, £40; wages, £55; coals, £18; gas, £15: repairs, £4; laundry, £30; total, £292.

The food consumed by housekeeper, two servants, and occasional helpers may be roughly set down as £60 to £80 per annum. All these expenses can in no way be met by the payments of a limited number of nurses.

### Everybody's Opinion.

[Correspondence on all subjects is invited, but we cannot in any way be responsible for the opinions expressed by our correspondents. No communications can be entertained if the name and address of the correspondent is not given, or unless one side of the paper only be written on.]

### THE PARISH NURSE.

"Veritas—One of the Queen's Nurses" writes: Your article in The Hospital for August 19th, entitled, "A New Order of General Practitioners," has, I should say, the hearty approval of every conscientious, practical nurse who does the work for which she has been trained, and does not presume to overstep that work and encroach on duties for which she has not been trained, and that properly belong to the medical practitioner in attendance, and under whose sole direction she nurses—or should nurse. I am alluding more particularly to the first part of the article *re* the parish nurse. I am not so well acquainted with the paying nursing homes, but with the parish nurse I have often come in contact, and I think if she were turned into a district visitor instead she would be of much more use, and do far less harm to the reputation of nurses (at least, amongst doctors). Now a good district nurse (*i.e.*, a nurse to nurse the sick poor in their own homes), thoroughly trained in hospital and afterwards at a recognised district nurses' institution, is invaluable to the doctors working amongst the poor; she undertakes the actual nursing, and leaves the doctor to give directions. The "parish nurse" is usually engaged by the clergyman, and works under his directions, supplemented by the curates, district visitors, and various ladies in the parish; she goes round taking beef-tea to one, puddings to another, giving directions as to the washing of helpless patients, opening of windows, prevention of bed-sores; if it is an acute case will take a temperature occasionally, and sometimes even give pills or simple mixtures. The former duties of taking round nourishment will be as well done by the district visitor; the latter ones will probably be done by the doctor. But if the nurse herself washed the patients, changed their linen, made the beds, attended to the state of backs, hips, &c., when there were inclinations to bed-sores, opened the windows, emptied the slops, in acute cases took the temperatures night and morning regularly, applied dressings, made beef-tea, &c., when necessary, and performed all those duties which a trained nurse should do, she would have to spend far more than ten minutes in each house; but then she would be of great use both to doctor and patient. Here is a typical case which occurred only a month ago: A woman dying of cancer of the breast was taken to one of the poor-law infirmaries by one of the district nurses working in South London. This nurse was only called in to prepare the patient for going to the infirmary; she was very neglected and begrimed with dirt; she had been visited by the parish nurse for nearly a year, who had just gone away for her holiday; but, as the patient's daughter remarked, "We got on just as well without her, as she never touched mother, and only came sometimes and talked." Now, if there had been no parish nurse in that particular parish this poor woman would have been properly cared for and attended during the whole course of her illness by a qualified district nurse. I remember a remark made by a Roman Catholic priest on the district nurses of one of the branches of the Queen's Nurses' Institute: "These nurses are not like women, they are more like angels; they come into the houses of the sick, they do their work and then they go; they do not talk and gossip." This priest evidently thought silence was gold; and though I see no harm in a little quiet talking with those patients not too ill to bear it whilst the various duties are being performed, still merely to go and talk or to stay and gossip is superfluous, and can be done equally well by untrained women. Nursing is a business, not a pastime; and unless there is actual nursing to be done, no case should be taken on by a parish or district nurse. Would a doctor send for a private nurse for his well-to-do patient merely to direct the patient's friends, and not to do the actual nursing herself? Decidedly not. The same holds good with the parish nurse, who, if she is well trained, will refuse to waste time and energy on work that is not within her province. What we want is a good supply of well-trained practical women who do their work quietly and thoroughly under their superior officers, viz., the doctors.

### CORNISH HOLIDAY RESORTS.

**"A Traveller" writes: To London nurses Cornwall seems a long way off, but the journey is a pleasant one, and the expense of it is balanced by the cheapness of lodgings and living at most of the places. There are Saturday excursion tickets issued to Penzance, which hold good for seventeen days, and cost much less than the ordinary return tickets. Lodgings can be obtained at about fourteen shillings a week for two rooms, and everything is spotlessly clean and attractive. There are numerous excursions to be made, none of which need be costly. At Newquay equally satisfactory accommodation is attainable, and Mr. Hartnell of this town publishes a list of apartments which is a great help to travellers. North Cornwall excursion tickets issued at Paddington are exceedingly moderate.**

### A NURSE'S HOME IN PARIS.

"A Matron" writes: I am delighted to see by your paper (August 19th) that Miss Woodcock intends to start a Nursing Home in Paris. I have never heard anything but praise of Miss Woodcock's Home, and wish her every success. I would earnestly advise nurses to be quite sure they are really joining this home, which will be conducted with regard to nurses' comfort, a most important matter when far from home.

### COTTAGE NURSES.

"A Member of the Dorset Health Association" writes: Referring to our short notice in the "Nursing Mirror" of August 12th on "The Work of the Dorset Health Association," I think that, in justice to the said society, it should be stated that 170 lectures on health were organised under its auspices last winter in the country under highly qualified lady lecturers of the National Health Society, not to mention those given under the St. John Ambulance, so that while trying in promoting organised cottage nursing to go a step further, yet they have not been unmindful of laying a firm foundation upon which to base their operations.

### The World of Nurses.

As seen by an Unprofessional Correspondent.

### II.—A CALLING OR A PROFESSION?

The payment of nurses is a vexed question, and it is difficult to know how their salaries can be increased, considering the poverty-stricken condition of the hospitals. But it is equally difficult to expect a woman to take to the life as a mere profession, when she, being a lady by birth, is remunerated at the rate of a cook. Even the much grumbling and much snubbed governess is better paid and less worked. Think of an educated and cultivated woman being worked twelve hours a-day for £30 to £40 a-year. What man is so remunerated? But then, you say, the mere calling compensates for the bad pay. Is that so? And is it fair to consume the best years of a woman's life in a profession which uses her up in ten or fifteen years, and pay her so little that it is almost impossible for her to save? If nurses are members of a sisterhood it is a different matter. They have given up the world; they devote themselves to their work as a labour of love; the money goes to the community, and, in case of illness or old age, the sisterhood provides for them. A sister of charity has no worldly goods, but she also has no cares either for the present or for the future. But the lay nurse has not given up her position in her family, and she knows that if she breaks up, she has no one to help her, and no means of helping herself unless she has private means. Surely, then, nurses ought to be better paid, and if so some means must be found to help the funds of hospitals instead of the endless begging. Now, it is well known that numbers of people go to the out-patient department of hospitals who could very well afford to pay a little, say a shilling for medicine. Often the poor consult a small general practitioner who charges a shilling or two; why, then, should not they pay when they prefer the advice of a hospital physician or surgeon? Would not these shillings help the funds of the hospital and also stop the grumbling so often heard from persons who are glad enough to take what they can get without paying for it?

But another plan also suggests itself. In Paris, I believe, certain members of the medical profession open a consulting-room for the poor middle classes, where they see patients for five francs. Now, surely something of this sort might be done at English hospitals? If the poor gentry want to consult an eminent physician or surgeon they have to get an introduction through a friend, and the medicine man—kind, good soul—and what numbers of such there are!) sees the patient and takes no fee. In no other profession is so much work done for nothing, and very often the thanks are as absent as the fees. But even when a patient is grateful there is an awkwardness about the matter; people do not care to be under an obligation to a doctor if they have any independence of character. Possibly, too, the fear of being placed under an obligation prevents the most right-minded from accepting a kindness which we know involves a certain loss of time and some trouble to another. Why then should not out-patients' departments be opened for paying patients—say, a fee of five shillings; it would swell the funds of institutions, and enable committees to raise the pay of the nurses, for it is probable that a large number of persons would avail themselves of such an advantage. It is utterly impossible for many men and most women who work for their living to give a fee of two guineas for a consultation, but they do not want to be under an obligation to the physician, and it is far better that they should pay something than receive advice gratis. Some such scheme as this would probably be of immense utility both to the public and the hospitals. Why should it not be tried?

Another matter that wants reform is the feeding department of some hospitals. Is it nice, is it palatable, that butter, bread, and such-like commodities, should be kept in a bed-room—nay, often in a small ward with a sick person? Even to those of us who live in a healthy atmosphere, I doubt whether we should care for butter which had been hours in a sick-room—the very idea of it is disagreeable.

All the griefs from which nurses suffer are simply and solely from want of sympathy on the part of the powers that be. Neither the members of the committee, nor the medical staff, nor the officials work for twelve hours on end for the sum of £20 to £40 a year. They none of them scramble over their dinners; they do not keep their sugar and butter in a locker in their bedroom; they do not sleep in dormitories or cubicles; nor are they upon the tramp all the livelong day. And yet these women, the weaker vessels of humanity, are supposed to be cheerful, good-tempered, active, contented, and "passing rich" on less than £40 a year! They have been called "angels," and verily, the position is so arranged that only angels could fill it properly. And why? Simply because a want of sympathy prevents the official staff from perceiving the difficulties, and an absence of imagination precludes them from putting themselves in the position of the nurse.

Considering the disabilities, it is a wonder so many really good and true women take to the calling. It is a high calling, and perhaps the most useful work a woman can do; but the nurses who look upon their profession from this point of view are those who are content to take the position as part payment for services, just as a clergyman does. These probably are in the minority. The majority look to the calling as a stepping-stone to worldly advancement; and to marriage as a compensation for hard work and poor pay. So let it be, only in this case let us cease to look upon a nurse as an angelic, devoted woman, full of self-sacrifice, and possessed by a desire to pass her life in doing good. Either one thing or the other. If angels, then let them cut themselves off from the world, and put aside towsled fringes, high heels, smart uniforms, general frivolity, and flirting; or if they affect all this, let them pose no longer as superior to the commonplace governess, the smart milliner, or the young maid of all work. There is just as much devotion and self-sacrifice in staying at home and putting up with bad tempers as in nursing, and if worldliness and worldly advancement are to be the rewards, one life is just as noble, or as contemptible, as the other. Why, then, make such a glamour over the woman who shirks the dull home life for the excitement of the hospital? But if the motive be really devotion, a life-long devotion to the cause, than the hospital is probably the more noble life. In the one case the self-sacrifice would probably lie in staying in the dull home; in the other, it would be in giving up the pleasures of this world, and submitting to training, the whole end and aim of which is to sacrifice self-will and all personal desires, and to become a servant of servants.

This is, perhaps, a high ideal for most women, but it is what is wanted in order to keep the calling from becoming a mere worldly profession. Let it be known that the true nurse is as much above the worldly woman in leading a life of self-sacrifice and devotion as the true priest is above the ordinary layman, and the ranks will soon be cleared of objectionable intruders. This is a matter for matrons.

But, while expecting a high tone of morals, and as pure unselfishness as is attainable in this life, there is no reason why the nurses should not be properly fed, and less improperly worked. This is a matter for the officials—let them put their houses in order. No sensible persons advocate an eight hours' day for any one, a forced eight hours only of work would be a penance to many; but there is a difference between eight and twelve or fourteen. Besides, it is just when a person is sick that some good, morally, might be done to the degraded. How can this be accomplished when the nurse has not a moment to expend on sympathy? Life in hospital is one perpetual "rush," and so many an opportunity of helping the distressed in mind as well as body is lost. Perhaps that side of the question may have more weight with the philanthropists who sit on committees and in comfortable homes than the weariness and semi-starvation of a few hundreds of devoted women. Saving a soul is generally thought more of than taking care of the body; but if the body be neglected, does it not endanger the soul by feeding ill-temper and general grumbling and bad feeling? Undoubtedly; but the soul of the patient is often that of a drunkard, a breaker of some moral law or other, perhaps of all the Ten Commandments put together, and so he is acutely interesting to the philanthropists; while the soul of the nurse is only that of a commonplace person who strives to do her duty in a plain-sailing, practical, commonplace fashion, because it is a duty, both to God and man.

Aihpos.

### Notes and Queries.

### Queries.

(176) *Hector* (*Perspiring Feet*).—Can you give me advice on this subject?

(177) (*Lady Nurses for Children*).—Can you give particulars of an institution where ladies can be trained as children's nurses?

(178) *E. A. W.* (*Sanitary Inspectors*).—I am anxious to know where women apply who wish to prepare for sanitary inspectors' work.

(179) *L. R.* (*Co-operation*).—Is there a Nursing Co-operation in Manchester or Liverpool?

(180) *N. S.* (*Book*).—Can anyone tell me of a good book on surgical appliances, with illustrations?

(181) *J. M.* (*Journal*).—Can you tell me if there is any publication in the United States equivalent to The Hospital? Could any of your correspondents give me information as to women's work, nursing, &c., in California?

### Answers.

(175) *Miss B.* (*Nursing Book*).—Write to the Manager, The Scientific Press, 428, Strand, for information.

(176) *Hector* (*Perspiring Feet*).—Washing the stockings in any way will not prevent. You should wash the feet daily twice, once with much-diluted Condy's Fluid, and once with a weak solution of alum. You should never wear stockings longer than a day, and it is best to keep two pairs in use, and change them during the day. Have you consulted a doctor?

(177) *Lady Nurses for Children*.—Write to Miss A. Coleman, Hospital and Home for Incurable Children, 2, Maida Vale, and to Mrs. Hilton's Crèche, 12, Stepney Causeway. There is also the Norland Institute, which trains.

(178) *E. A. W.* (*Sanitary Inspectors*).—Write for the prospectus and past examination papers of the Sanitary Institute, 74a, Margaret Street, W., and write to Miss Lamport, 155, Burton Crescent, W.C., who would doubt ess advise you as to coaching.

(179) *L. R.* (*Co-operation*).—We do not know of any Nurses' Co-operation in Manchester or Liverpool. A list of nursing institutions is given in "Burdett's Hospital Annual," 428, Strand.

(180) *N. S.* (*Book*).—A series of articles such as you want appeared in our columns during 1891. These are to be shortly published, with additions, in book form, by The Scientific Press, 428, Strand. The book will supply a general want.

(181) *J. M.* (*Journal*).—There is no American paper on the lines of The Hospital. The *Trained Nurse*, 14, Veysey Street, New York, published monthly, is the best nursing paper.

### For Reading to the Sick.

### THE FAINT HEART.

"Do not weep for me," said a sick man to his friends, who were standing round his bed deploring his sickness and agony, "Do not wish away my pain, but wish that in all my griefs, I may have a wise and thankful heart, firmly staged on God, and never starting aside from the loving correction He sends me." Suffering had wrought a blessed work in that man's soul. He had, probably after "many a struggle, many a groan and many a tear," resigned himself into his Heavenly Father's hands to do with him as He would. He had learnt that it was the better part, not to toil and fret in an ever restless desire for ease, but to let him strike home and meekly lie still and kiss the rod. Ah, we are never so safe or so happy as when our will yields itself to God without stint or fear. It is the sweetest offering we can make to Him who gave for us and to us the "only son of His dear love."

Why, then, is it that the souls of men, that our own souls, do not take this comfort to themselves, but let ourselves be scattered about like a crowd of frightened sheep seeking it where it cannot be found? Why do our foolish hearts wander from a love so true and deep, when our Saviour would have us gather around Him and cast all our burdens on Him? Are our hearts faint? Do we fear to trust all to Him? We have heard God's love is mighty, yes, far mightier than it seems to us who give so little in return. We have been told His mercy is so wide, hear what the Psalmist says, "Look how high the heaven is in comparison of the earth, so great is His mercy also towards them that fear Him. Look how far the east is from the west, so far hath He set our sins from us." He does not deal with us after our sins, nor reward us according to our wickedness, but as a father pitieth his children the Lord is merciful to them that fear Him.

We must not think, because our blessed Lord has left the earth, He is so far from us that He cares nothing for those He died to save. Though He is now in heaven sitting on the right hand of God, He feels for all our sorrows, He is always pleading His death with the Father for the pardon of our sins, showing His pierced hands, His riven side, "those dear tokens of His passion," those wounds which were for the healing of the nations. Let us, then, take courage and come boldly to the throne of grace. God's love has no limits but what we, in our fear, make for it. At the feet of Jesus—

There is welcome for the sinner,
And more graces for the good;
There is mercy with the Saviour,
There is healing in His blood.

There is plentiful redemption
In the blood that has been shed;
There is joy for all the members
In the sorrows of the Head.

### Minor Appointment.

Rochdale Infirmary.—Miss Rothwell was appointed Matron of this infirmary on August 18th. She was trained at the Manchester Southern Hospital and the Victoria Hospital, Chelsea. Subsequently Miss Rothwell had charge of the accident ward at the Wolverhampton General Hospital, and later filled the post of Head Nurse at the North Riding Infirmary, Middlesborough. In this capacity she remained for three years. She then was elected Matron of the Eston Hospital, Middlesborough, which post she filled for nine years. Miss Rothwell goes to her new appointment with the good wishes of all amongst whom she has worked. Her testimonials are most excellent.

### Wants and Workers.

A lady would be glad to hear of a comfortable home for highly-respectable old nurse. She is capable of performing light duties, and is good needlewoman and an excellent nurse. A small salary only required, a comfortable home being the main object. Address replies to the Editor of The Hospital, The Lodge, Porchester Square, W

### The Muses' Looking Glass.

### AN INTERNATIONAL KISS.

They have everything at Chicago this year. In their universality they have gone so far, these democratic Americans, as to import specimens of the effete aristocracy and played-out royalties of Europe. The specimens are Spanish, for America is nothing if not Spanish to-day, and if it admits any other ancestry than that of Columbus and his followers, it is to claim descent from the hardy Vikings whom myth and legend declare to have crossed the Atlantic in an open boat about the time that Thor and Odin were building Valhalla.

First the managers of the show got hold of the Duke of Veragua, a lineal descendent, so it is averred, of Christopher Columbus. This was good; but a greater than Veragua was coming. It was announced that the Infanta Maria Eulalia of Spain, the lineal descendant of that Isabella who was the patroness of the great Genoese, was about to visit America. America went mad with the excitement of the thought. The Infanta's husband, who is also her cousin, Don Antonio de Bourbon, was coming too, but nobody bothered about him. But Eulalia was coming, and it was resolved to do her all honour. Unfortunately, the Great Republicans, not being used to princesses, were not quite sure how the honour was to be paid. There are certain frivolous, meaningless, despicable rules of etiquette in courts which the free-born American despises as a rule; but it is convenient to know them when a princess comes to see you. And it is worse with a Spanish princess than any other; for the etiquette of Spain transcends all other in complexity and stiffness. There when a beggar meets a fellow tramp he addresses him as "Senor Caballero," and it is said that the real reason why the late Duke of Aosta was forced to renounce the Spanish crown he had accepted was that he roused the indignation of his nobles through inadvertently addressing his valet in giving an order by the familiar "thou" common in Latin lands. In Italy or France it would not have mattered much; but in Spain the Royal *tutoiement* transformed the valet into a peer, and the blue blood of Castile could not brook the intrusion, so the valet was dismissed—or assassinated—and the king was deposed.

In that aristocratic land so much depends on the inflection of monosyllable that angels might err, or even Americans, and fears were expressed that some chance tone of the voice, some omission of a bow or rash assumption of a seat, might give the Infanta cause to think she was treated like a laundress or a cook. True it is that in the course of a fourteen-column article on the subject a newspaper said: "The Infanta Eulalia will be treated with an etiquette that transcends the rules of any Court on earth. She will receive the respectful admiration of a free and enlightened nation, and she will command it by her personality, not by her rank." That is very pretty; but it doesn't tell you whether to shake hands and say "How do you do?" or to bow to the ground with "May it please your Royal Highness."

For one thing, it is usual, in this despised etiquette of Courts, for kings and queens to kiss each other when they meet, addressing each other as "my brother" and "my sister." Was it President Cleveland's duty, as head of a nation greater than any empire, to kiss the Infanta Eulalia? A continent was convulsed with the mighty problem. For our own part, we think it might safely have been left to Mrs. Cleveland to settle. If she didn't mind her husband kissing another young and charming lady, why should anybody else? It is true that Don Antonio might have an opinion to offer; but, as has been said, one didn't hear much about him, and perhaps it was assumed that Eulalia would not really want to kiss "Grover," though she might consent to his embrace as a matter of duty. The newspapers discussed the matter *pro* and *con*, but, on the whole, their opinion seemed to be that it is well to kiss a pretty woman whenever you get the chance. Prominent men were asked their opinion, and almost to a man they recommended the kiss. Mr. John C. Calhoun declared himself "a warm adherent of the chaste salute on the lips, whether it be a princess or a simple country maid," but he admitted that in view of "certain existing conditions," meaning that both the princess and the President were both married, the "official osculation" should take place in the presence of the near relatives and intimate friends of the two families. (Query: Is a kiss worth having under the circumstances?) Probably Colonel Boykin Ford meant the same thing when he advised the President to kiss the lady in an "abstract, informal, and diplomatic fashion." "There are kisses and there are kisses, I am told," says he. Wise yet innocent Colonel! Evidently he knows from experience only one kind of kiss, probably neither abstract nor diplomatic. Free thought and orthodoxy are at one upon the kiss. Colonel Ingersoll, indeed, after some appreciative remarks on kissing in general, pulls himself up just as he is about to give some practical advice with the reflection that he is a family man, but it is evident that his sympathies are osculatory; while Dr. Talmage, though he "hopes he won't do it," admits that he "didn't know of any moral law or any law of the land to prevent President Cleveland from kissing the Infanta either upon the mouth or the hand." A notable police magistrate, Mr. Barney Martin, put in his word: "If I were President and a good-looking girl came along from Rooshia or Africa, or any of them foreign places, you can bet yer sweet life I'd kiss her every time."

This was frank enough; but, after all, a police magistrate cannot be expected to decide authoritatively on a point of international manners as if it were a case of "drunk and disorderly," and it was universally felt that the right thing had been done when it was resolved to propound the conundrum to Ward McAlister. Ward McAlister is the acknowledged leader of society in America, of that select Four Hundred compared with whom our Upper Ten Thousand are but a vulgar mob. What McAlister doesn't know about cotillion favours isn't worth knowing, and his definition of the lines of the ideal "claw-hammer," coat is acknowledged to be masterly, even by tailors. But when interviewed on the subject of Eulalia and the kiss, he owned sadly that he didn't know what the President ought to do. The confession was printed in letters half an inch long, and the American consciousness was as far from rest as ever.

It did not seem to have occurred to anyone that the question might have been settled by the Infanta herself; and that it would have been well to ask her if she wanted to be kissed by the President of the United States. Ladies have their prejudices in these matters. But is it not a little strange that in a country of so much earnestness and energy, a country of great ambitions and practical ideals, they have time to fill the columns of their newspapers with questions which to us of old snobbish, toadying, monarchic Europe seem to be unworthy of a moment's consideration? Are our big cousins only children after all?

### Nurses and Cholera.

Ireland has adopted its own method of meeting the possible emergency of an outbreak of cholera and the attendant demand for nurses. We learn that a Dublin hospital has opened its doors to six probationers, free of charge, who will receive instruction and have their names placed first for engagement should the epidemic occur. Limerick, following suit, advertised the same advantages, but at a meeting of the Board of Guardians it was stated that no applications had been received; the offer, however, is to be continued for some time. We can only hope that the hospitals will not find themselves sorely taxed by the doubtful advantage of an invasion of probationers. It is doubtful whether the lessened work in the wards for regular nurses compensates for the trials of temper caused by the raw probationer.

### The Book World for Women and Nurses.

[We invite Correspondence, Criticism, Enquiries, and Notes on Books likely to interest Women and Nurses. Address, Editor, The Hospital (Nurses' Book World), 428, Strand, W.C.]

### "NEITHER FAITHFUL NOR POETICAL."

Lyrics and Ballads of Heine and other German Poets. Translated by Frances Hellman. (New York: G. P. Putnam's Sons, 1892.)

It is not given to everyone to go to Corinth. Even in these days, when education is constantly spreading wider, there are still many who thirst after an acquaintance with the masterpieces of foreign minds, but must be content to acquire it at second hand. The demand, no doubt, creates the supply, and every year sees the works of non-English authors dished up, or re-dished up, in English for the benefit of the British reader. On this we have only to congratulate ourselves, and our thanks are distinctly due to the army of translators who bring the best works of Continental writers before us in a form to be understanded of the people. And where prose is concerned, there is really very little to complain of. It may be granted that the Englishing of French, German, Russian, Italian, Spanish, and Scandinavian novelists, playwrights, philosophers, and historians is on an average fairly well done. The renderers may claim at least that they reflect faithfully the language of their author, and, at the same time, avoid outraging their own.

When we come to Poetry, however, it is impossible to pass so favourable a general judgment. We do not here refer to those modest translators who are content to turn exotic poetry into native prose. Such lack of ambition is laudable, but rare. It is when the translator aims higher, and would fain present the foreign poet in the garb of English verse that disasters multiply. The aim is a perfectly legitimate one, and success would deservedly bring with it laud and honour. But the genius which can command such success is a plant of rare growth, and, when it fails to attain perfection, is a poor vegetable indeed. Such translations may be classified by their results under four heads: (1) the Faithful but Prosy; (2) the Paraphrastic but Poetical; (3) the Faithful and Poetical; and (4) the neither Faithful nor Poetical. Class 1 is almost too common to require exemplification, but Milton's rendering of "Quis multa gracilis te, puer, in rosa" may serve as an awful warning against publishing schoolboy effusions. Of class 2 Thackeray's translations from Béranger furnish examples, and several of Longfellow's might be cited in this connection. Class 3, which is necessarily the rarest and most admirable, may be aptly illustrated by Swinburne's well-nigh faultless version of "Où sont les neiges d'antan?" Of class 4 the work under review affords a perfect specimen.

We are not in Mrs. Hellman's confidence, and have no idea what impelled her to submit these translations to the public, nor are we aware on what terms the Knickerbocker Press undertook their publication. In the absence of data we diffidently conjecture that Mrs. Hellman began by translating a poem or two, culled here and there from the German anthology. Injudicious friends probably pronounced the translations to be very, very good, and further went on to wonder why she did not transmute the whole of Heine's lyrics into English poetry and then publish the lot. On this theory we should assume that the nucleus of the present volume was formed by the authoress's translation of

THE BOOK WORLD FOR WOMEN AND NURSES—*continued.*

Freiligrath's *O lieb so lang du lieben kannst,* which really runs with some grace and smoothness :

"Oh ! love as long as thou canst love !
Oh ! love as long as love will last !
The hour will come, the hour will come,
When, over graves, thou'llt mourn the past."
(p. 227.)

Possibly the reception of her renderings of Goethe's *Der Wand'rer's Nachtlied, Meeresstille* and *Glückliche Fahrt* (pp. 145, 146, 147) also had their share in encouraging her to venture further afield. It was a bold undertaking to try the hand on *Uber allen Gipfeln ist Ruh,* that most untranslatable of tiny poems, and she has only succeeded partially and at the expense of two lines more than the original warrants. In *Gluckliche Fahrt,* though the *allegro* idea is not unhappily reproduced, we must protest against "The anxious band" as a translation of *Das ängstliche Band.* In justice to Mrs. Hellman we admit that she is not often guilty of similar solecisms, though we have detected a few here and there. On p. 80, for instance, in the version of *Ich stand in dunkeln Fraumen,* the word *heimlich,* which here means "mysterious," is wrongly rendered "secret." Again, at p. 75, in the fourth line of verse 1, "whisper" is scarcely a correct equivalent for *kosen.*

It is not of these occasional trifling inaccuracies that we complain. The authoress might fairly plead the exigencies of metre and earn the indulgence of the critic. But we can find no excuse whatever for the terrible bathos of too many passages—the puerility and lack of dignity with which she has invested the lines of these standard poets of Germany while essaying to endue them with an English garb. Nothing could well exceed in feebleness the following examples, which might, unfortunately, easily be multiplied :—

"At morn I rise and query :
Will Sweetheart come to-day ?
At night I sink down weary,
'Again she's stayed away.'" (p. 13.)

Englishmen do not "query" in common life unless they propose to question the accuracy of some point ; nor do they use the word "sweetheart" absolutely, except in the vocative case.

"In bright and tiny medley,
Appear on yonder side,
The villas, and gardens, and people,
Woods, oxen, and meadows wide." (p. 73.)

A medley, indeed ! but why "tiny"? "Lessened by distance" is doubtless the meaning sought to be conveyed, but then it is not conveyed, and the unfortunate German poet is wronged, without having done anything to the translator to warrant such treatment.

We will conclude our extracts with the last verse of the "Lorelei," which exhibits both Mrs. Hellman's lack of appreciation of the original, and her want of skill in the construction of the English verse :—

"Methinks the waves will have swallowed
Both boat and boatman anon ;
And this, with her song unhallowed,
The Lorely hath done." (p. 72.)

We are ready to allow that the plea of extreme difficulty may be advanced by the translator as an explanation of the appallingly slipshod result. What we do not admit is that Mrs. Hellman was in any way called upon to grapple with a task of this magnitude. Where the acknowledged poets of England and America have too often failed, it is no disgrace to lesser lights to fall short. But they should keep their shortcomings private, and not flaunt them before the public gaze.

